# Background factors of chemical intolerance and parent–child relationships

**DOI:** 10.1186/s12199-018-0743-y

**Published:** 2018-10-24

**Authors:** Kenichi Azuma, Masayuki Ohyama, Emiko Azuma, Takae Nakajima

**Affiliations:** 1 Department of Environmental Medicine and Behavioral Science, Kindai University Faculty of Medicine, 377-2 Ohnohigashi, Osakasayama, 589-8511 Japan; 2 Department of Environmental Health, Osaka Institute of Public Health, Osaka, 537-0025 Japan; 3 Department of Food Chemistry, Osaka Institute of Public Health, Osaka, 537-0025 Japan

**Keywords:** Allergy, Asthma, Chemical intolerance, Childhood, Cold sensitivity, Indoor pet

## Abstract

**Background:**

Chemical intolerance is a widespread public health problem characterized by symptoms that reportedly result from low-level exposure to chemicals. Although several studies have reported factors related to chemical intolerance in adults, the impact of family members has not been reported. In the present study, we investigated the background factors related to chemical intolerance in family members and parent–child relationships.

**Methods:**

We distributed a self-reported questionnaire to 4325 mothers who were invited to visit the Kishiwada Health Center in Kishiwada City, Osaka, between January 2006 and December 2007 for the regular health checkup of their three-and-a-half-year-old children.

**Results:**

The prevalence of chemical intolerance in the 3-year-old children was almost one eighteenth of that reported by their mothers. Multiple logistic regression analyses revealed that cold sensitivity [odds ratio (OR), 1.89; 95% confidence interval (CI), 1.04–3.44], past bronchial asthma (OR, 2.84; 95% CI, 1.46–5.53), and any past allergies (OR, 2.21; 95% CI, 1.36–3.60) were significantly associated with chemical intolerance in the mother. The presence of indoor cat during childhood (OR, 1.99; 95% CI, 1.08–3.69) was significantly associated with chemical intolerance in the mother; however, the association was weak compared with cold sensitivity and past asthma and allergies. The current chemical intolerance of the mother was significantly associated with allergic rhinitis (OR, 2.32; 95% CI, 1.19–4.53), bronchial asthma (OR, 3.66; 95% CI, 2.00–6.69), and chronic bronchitis (OR, 3.69; 95% CI, 1.04–13.03) in her 3-year-old child.

**Conclusions:**

The results suggest that inherent physical constitution and childhood housing environment are associated with a risk of acquiring chemical intolerance. Children of mothers with chemical intolerance have a possible risk of respiratory hypersensitivity or inflammation. Further investigation is recommended to determine the inherent physical constitution and background environmental factors associated with the risk of acquiring chemical intolerance. The impact of having mothers with chemical intolerance on the health of children also requires further study.

**Electronic supplementary material:**

The online version of this article (10.1186/s12199-018-0743-y) contains supplementary material, which is available to authorized users.

## Background

Between 8 and 33% of people in various populations consider themselves to be unusually sensitive to odorous and pungent chemicals, with the variability in prevalence depending largely on a wide variety of definitions and severity. This condition, known as chemical intolerance (CI), is a frequently reported problem in industrialized countries [1–9]. Multiple chemical sensitivity (MCS) and idiopathic environmental intolerance (IEI) are labels often used for severe cases of CI.

CI is a chronic acquired disorder characterized by non-specific and recurrent symptoms in multiple organ systems associated with exposure to low levels of odorous chemicals such as organic solvents, pesticides, perfumes, cleaning products, perfumes, tobacco smoke, car exhaust, or combustion products [10–12]. Despite its relatively high prevalence in nonclinical samples, the diagnosis and etiology of CI remain controversial and understudied. Although the mechanisms behind CI are unknown, various theories highlight that alterations in chemical sensory transduction and neural processing, rather than toxic processes, serve as key components [13–18].

Phenomenologically, the development of CI appears to be associated with a loss of tolerance following acute or chronic exposure to various environmental agents in daily life and the subsequent triggering of symptoms by extremely small quantities of previously tolerated chemicals [19]. Although the effect of age on CI is unclear, the prevalence of CI in persons ≥ 60 years of age is lower than that in other age groups [2, 4] and the prevalence of CI in teenagers is much lower than that in adults [20]. In addition, the importance of genetic factors involved in xenobiotic metabolism in CI etiology is still uncertain concerning the importance of genetic variability as opposed to epigenetic mutation [16, 21]. Although the underlying mechanisms between asthma/allergy and CI may be different, several studies reported comorbidity and multimorbidity between these conditions [22–24]. Therefore, as the medical history of the family and the housing environment during prenatal and early life periods are possibly associated with the development of asthma and allergies [25–27], similar associations may be seen in CI. Clarifying the environmental factors or predisposing causes associated with CI during these periods would enhance our understanding of CI and assist in the development of future strategies to prevent it. Considering those insights, the aim of this study was to investigate the background factors in family members and parent–child relationships that may influence the development of CI.

## Methods

### Study design and population

A cross-sectional design was employed in this study. In accordance with Article 12 of the Maternal and Child Health Act in Japan, the regular health checkups for all children at 18 months and 3 years of age are provided by the municipality. A questionnaire was distributed to all mothers who were invited to visit the Kishiwada Health Center in Kishiwada City to undergo the regular health checkup of their three-and-a-half-year-old children between January 2006 and December 2007. The questionnaire was included in an invitation letter for the health checkup that the Kishiwada Health Center distributed to all candidate mothers during the 2 years. The questionnaires were collected when the mothers visited the Kishiwada Health Center.

Kishiwada is a city located in the southern area of Osaka Prefecture and is one of the municipalities of Japan. In April 2017, the city had an estimated population of 200,000 people and a population density of about 2700 persons per square kilometer. The city’s total area is 72.68 km^2^. Kishiwada is one of the central cites in Osaka Prefecture.

### Questionnaires

All responses were self-reported by the mothers responding to the survey. The questionnaire included items about the mother regarding demographics, medical history of the family, CI including children, physical constitution, dietary habits, psychosomatic state during the past month (nervousness, peacefulness, depressed feelings, happiness, and discouragement), birth order of children, and housing environment as an elementary school-aged child (type of housing, floor material, insecticide applications, and indoor pets in the first decade of life). The questions on medical history addressed hay fever, allergic rhinitis, allergic conjunctivitis, alimentary allergy, medication allergy, atopic dermatitis, bronchial asthma, empyema, sinusitis, chronic bronchitis, rheumatoid arthritis, gastric ulcer, MCS, and sick building syndrome.

To identify the presence of CI, several questionnaires with self-reported responses have been developed for research purposes [7, 28–32]. Some questionnaires focused only on one domain, such as the number of odorous chemical exposures that elicited symptoms or the frequency with which specific chemical odors caused illness, and some had many questions with a more broad perspective. Black et al. developed a questionnaire [28] to identify CI that included standardized questions and scales to enhance the validity and generalizability of the results. The multisystem symptoms, multiple intolerances, and multiple behavioral changes often associated with CI were considered in the questions. This questionnaire was used in another recent study [33]; therefore, it was used in the current study to identify CI.

The questionnaire consisted of four parts: (1) one main question on the chemical sensitivity (routine or normal levels of exposure to chemical agents/substances such as gasoline, hair spray, paint, perfume, and soap caused respondent to feel ill) and three domains related to CI; (2) sensitivity to 15 incitants (1, vehicle exhaust/fumes; 2, smog/air pollution; 3, cigarette smoke; 4, copiers, printers, and office machines; 5, newsprint; 6, pesticides, herbicides, and fertilizers; 7, new buildings; 8, carpeting and drapery; 9, organic chemicals, solvents, glues, paints, and fuel; 10, cosmetics, perfumes, hair spray, deodorants, and nail polish; and our study added 11, mold odor; 12, disinfectants, bleaches, and detergents; 13, asphalt and coal tar; 14, furniture; and 15, do-it-yourself stores, footwear departments, and automotive interiors, in accordance with the Japanese lifestyle); (3) 10 symptoms in organ systems (1, constitutional [fever, night sweats, fatigue, weight loss, and weight gain]; 2, rheumatologic; 3, cardiovascular; 4, gastroenterologic; 5, dermatologic; 6, pulmonary; 7, cognitive; and neurologic symptoms, which were divided into 8, dizziness, postural hypotension, and numbness in a limb and 9, headache; and our study added 10, ocular, in accordance with Japanese situations); and (4) five behavioral changes in response to the perceived sensitivity (1, wearing mask, gloves, or special clothes; 2, changing one’s lifestyle to minimize chemical exposure; 3, moving to a new home/location; 4, use of special vitamins, supplements, or diets; and 5, use of oxygen, antifungal agents, or neutralizing injections/drops).

### Criteria for identifying chemical intolerance

Black et al. included the criteria to identify CI in their questionnaire [28]. In their study, a panel of physicians who were experts in allergy, immunology, occupational health, environmental health, clinical epidemiology, and psychiatry developed an operational definition for CI based on a review of existing case definitions and their experience. The criteria required that a person reported the experience of illness from chemical sensitivity, reported sensitivity to two or more types of incitants, had symptoms in at least two organ systems, and had one or more behavioral changes in response to the perceived sensitivity [28].

### Statistical analyses

Correlations among the variables (medical history, housing environment, and psychosomatic state) were examined for multicollinearity by creating a correlation matrix and scanning for highly correlated variables (≥ 0.5). To prevent multicollinearity, highly correlated variables were not included in the multiple logistic regression model [34, 35]. Univariate associations were examined to identify the relationship between CI and potential relative factors.

In mothers, possible personal factors and past medical history related to CI were tested in multiple logistic regression analyses to determine possible predisposing causes associated with CI. Possible confounders were selected by reviewing the literature. Analysis adjusted for identified confounders, including age, employment status, and smoking status [1, 2, 4, 16] (model 1). Second, the association between CI and past housing environment was analyzed to determine a likely association with CI, adjusting for the possible confounders (model 2). Additionally, based on model 1 (six allergic symptoms integrated into “any allergic symptoms” to ensure stable statistical power), the past housing environment and psychosomatic state during the past month were tested to identify independent risk factors for CI (model 3).

In addition, to identify the characteristics of health status in children of mothers with CI, associations between the medical history of the 3-year-old child and the CI of the mother were analyzed, adjusting for the gender and birth order of the child, age and employment status of the mother, and drinking habits and smoking history of the mother. *P* < 0.05 indicated statistical significance. Odds ratio (OR) and 95% confidence interval (CI) were determined for the univariate and multivariable associations. All data analyses were performed using SPSS statistics software, version 23 (IBM Corp, Armonk, NY, USA).

## Results

### Participants

Of the 4325 mothers who received an invitation, 2044 responded (response rate, 47.3%). The participant characteristics of the mothers and 3-year-old children are shown in Table 1. The mean age of the mothers was 32.9 years (range, 18–47 years), and most participants were homemakers. Of the children, 47.5% were female and 49.8% were first births. In the physical constitutions of the mothers, 69.3% had cold sensitivity and 37.1% had sweatiness.

**Table 1:** Characteristics of mothers and their 3-year-old children (*N* = 2044)

	*n*/*N* (%)^a^
Mothers	
Age group	
18–29	424/1990 (21.3)
30–39	1436/1990 (72.2)
40–49	130/1990 (6.5)
Body mass index	
Low weight	326/1894 (17.2)
Normal weight	1399/1894 (73.9)
Obesity	169/1894 (8.9)
Employment	
Homemaker	1226/2015 (60.8)
Part-time employed	414/2015 (20.5)
Employed	362/2015 (18.0)
Others	13/2015 (0.6)
Smoking history (with)	664/2014 (33.0)
Drinking habits	
Never	1244/1992 (62.4)
1–3 days a week	457/1992 (22.9)
4–6 days a week	158/1992 (7.9)
Everyday	133/1992 (6.7)
Regular exercise (with)	517/2044 (25.3)
Eating regular meals (with)	1734/2022 (85.8)
Sweaty (with)	743/2002 (37.1)
Cold sensitivity (with)	1390/2005 (69.3)
Three-year-old children	
Gender	
Male	1067/2033 (52.5)
Female	966/2033 (47.5)
Birth order	
First	1000/2007 (49.8)
Second or after	1007/2007 (50.2)

^a^Data for some characteristics were missing for some participants

### Prevalence of chemical intolerance

Table 2 shows the prevalence of current CI estimated from the four criteria defined by Black et al. [28] and past medical history. The prevalence of current CI in mothers and 3-year-old children was 4.5% and 0.25%, respectively. The most prevalent past illness was allergic rhinitis (20.7%) in mothers, hay fever (12.4%) in fathers, and alimentary allergy (11.6%) in the children. The prevalence of current CI in the children was very low, so the analyses on this symptom were presented as supplementary data in subsequent modeling because of lack of statistical power.

**Table 2:** Prevalence of current chemical intolerance and past medical history (*N* = 2044)

	Mother *n*/*N* (%)^a^	Father *n*/*N* (%)^a^	Three-year-old child *n*/*N* (%)^a^
Chemical intolerance	91/2028 (4.5)	n.a.	5/1989 (0.25)
Allergies			
Hay fever	401/2044 (19.6)	254/2044 (12.4)	21/2043 (1.0)
Allergic rhinitis	423/2044 (20.7)	184/2044 (9.0)	125/2043 (6.1)
Allergic conjunctivitis	237/2044 (11.6)	57/2044 (2.8)	50/2043 (2.4)
Alimentary allergy	41/2044 (2.0)	22/2044 (1.1)	238/2043 (11.6)
Medication allergy	39/2044 (1.9)	9/2044 (0.4)	17/2043 (0.8)
Atopic dermatitis	164/2044 (8.0)	80/2044 (3.9)	202/2043 (9.9)
Any allergies^b^	792/2044 (38.7)	452/2044 (22.1)	472/2043 (23.1)
Bronchial asthma	105/2044 (5.1)	86/2044 (4.2)	139/2043 (6.8)
Empyema, sinusitis	221/2044 (10.8)	121/2044 (5.9)	96/2043 (4.7)
Chronic bronchitis	15/2044 (0.7)	9/2044 (0.4)	22/2043 (1.1)
Rheumatoid arthritis	5/2044 (0.2)	2/2044 (0.1)	0/2043 (0.0)
Gastric ulcer	43/2044 (2.1)	61/2044 (3.0)	0/2043 (0.0)
Multiple chemical sensitivity	0/2044 (0.0)	0/2044 (0.0)	1/2043 (0.05)
Sick building syndrome	3/2044 (0.1)	2/2044 (0.1)	2/2043 (0.1)

^a^Data for some characteristics were missing for some participants. ^b^At least one of six allergies (hay fever, allergic rhinitis, allergic conjunctivitis, alimentary allergy, medication allergy, atopic dermatitis) reported by participants. *n.a.* not applicable

### Background factors associated with chemical intolerance in mothers

Correlations among the variables (medical history, housing environment, and psychosomatic state) were examined. In the psychosomatic state, feelings of happiness and discouragement showed high correlation with peacefulness and depressed feelings, respectively. Nervousness showed high correlation with depressed feelings and discouragement and had a negative correlation with peacefulness. Nervousness had a strong association with CI in univariate analysis. Therefore, nervousness was included in the multivariable model. The univariate associations between current CI in mothers and all personal and other variables are listed in Table 3. The subsequent results of the multiple logistic regression analyses for the association with the CI are shown in Table 4.

**Table 3:** Univariate analysis for the chemical intolerance of mothers

Variables	Crude OR (95% CI)
Personal factors	
Age	
18–29	Ref.
30–39	1.18 (0.68–2.07)
40–49	1.69 (0.71–4.05)
*p* for trend	0.497
Body mass index	
Low weight	Ref.
Normal weight	0.81 (0.47–1.41)
Obesity	1.02 (0.44–2.34)
*p* for trend	0.672
Employment	
Homemaker	Ref.
Part-time employed	1.39 (0.82–2.35)
Employed	1.59 (0.94–2.70)
Others	–
Smoking history (with)	0.89 (0.56–1.41)
Drinking habits	
Never	Ref.
1–3 days a week	1.24 (0.76–2.03)
4–6 days a week	1.20 (0.56–2.57)
Everyday	0.35 (0.08–1.43)
*p* for trend	0.348
Regular exercise (with)	0.88 (0.54–1.45)
Eating regular meals (with)	0.56 (0.33–0.93)*
Sweaty (with)	1.33 (0.86–2.04)
Cold sensitivity (with)	2.26 (1.29–3.97)**
Past medical history (mother)	
Allergies	
Hay fever	2.99 (1.94–4.62)***
Allergic rhinitis	2.78 (1.80–4.29)***
Allergic conjunctivitis	2.57 (1.56–4.23)***
Alimentary allergy	3.84 (1.57–9.37)**
Medication allergy	1.80 (0.54–5.96)
Atopic dermatitis	2.66 (1.51–4.68)***
Any allergies^b^	3.07 (1.98–4.77)***
Bronchial asthma	4.05 (2.24–7.33)***
Empyema, sinusitis	1.54 (0.85–2.76)
Chronic bronchitis	1.64 (0.21–12.71)
Rheumatoid arthritis	–
Gastric ulcer	2.29 (0.80–6.58)
Multiple chemical sensitivity	–
Sick building syndrome	–
Housing environment in elementary school-aged child	
Type of housing	
Reinforced	Ref.
Wooden	1.43 (0.83–2.44)
Combined with reinforced and wooden	2.13 (0.26–17.24)
Floor material	
Wood	1.15 (0.75–1.78)
Japanese tatami	1.30 (0.77–2.20)
Carpet	1.25 (0.82–1.90)
Plastics	1.28 (0.51–3.24)
Spray of pesticide indoors (with)	1.52 (0.94–2.45)
Use of mosquito coil indoors (with)	2.09 (0.90–4.84)
Indoor pet parenting in the first decade of life	
Dog	1.51 (0.91–2.52)
Cat	2.23 (1.27–3.92)**
Housing environment in the present	
Indoor pet parenting (current)	
Dog	1.72 (0.94–3.16)
Cat	0.75 (0.74–4.14)
Psychosomatic state past month^a,b^	
Nervous	1.40 (1.21–1.62)***
Peaceful	0.83 (0.71–0.96)*
Depressed feeling	1.27 (1.08–1.49)**
Happiness feeling	0.86 (0.74–1.00)
Discouraged	1.20 (0.99–1.44)

Values are expressed as crude odds ratios (95% CI) for 2044 participants with complete data. Ref. = referent. Significant at *p < 0.05, **p < 0.01, ***p < 0.001. Text in parentheses reflects case groups. ^a^ORs and 95% CIs for six-level linear variables were calculated using one unit of change. ^b^The six levels were (1) not at all (0%), (2) slight (20%), (3) somewhat (40%), (4) moderate (60%), (5) often (80%), and (6) always (100%). Horizontal lines were expressed as the variable not analyzed due to the small numbers

**Table 4:** Multivariable analysis for the chemical intolerance of mothers

Variables	Model 1	Model 2	Model 3
Personal factors			
Eating regular meals (with)	0.50 (0.28–0.89)*		0.50 (0.28–0.91)*
Cold sensitivity (with)	2.00 (1.11–3.61)*		1.89 (1.04–3.44)*
Past medical history (mother)			
Allergies			
Hay fever	1.82 (1.08–3.08)*		
Allergic rhinitis	1.41 (0.81–2.47)		
Allergic conjunctivitis	1.18 (0.63–2.22)		
Alimentary allergy	2.11 (0.74–6.01)		
Atopic dermatitis	1.91 (0.99–3.67)		
Any allergies^b^			2.21 (1.36–3.60)**
Bronchial asthma	2.50 (1.26–4.98)**		2.84 (1.46–5.53)**
Gastric ulcer	2.06 (0.68–6.20)		1.67 (0.54–5.17)
Housing environment in elementary school-aged child			
Spray of pesticide indoors (with)		1.54 (0.93–2.55)	1.27 (0.76–2.14)
Use of mosquito coil indoors (with)		1.06 (0.45–2.51)	1.10 (0.45–2.67)
Indoor pets in the first decade of life			
Dog		1.30 (0.75–2.26)	1.22 (0.69–2.16)
Cat		1.99 (1.08–3.69)*	1.80 (0.95–3.41)
Psychosomatic state past month^a,b^			
Nervous			1.25 (1.06–1.48)**

Values are expressed as adjusted odds ratios (95% confidential interval) for 2044 participants with complete data. Significant at *p < 0.05, **p < 0.01. The predictor variables of chemical intolerance of mothers were selected from possible risk factors, possible confounders, and multicollinearity test (*r* < 0.5). Model 1: personal factors (eating regular meals, cold sensitivity, age, employment status, and smoking status) and past medical history; Model 2: personal factors (age, employment status, and smoking status) and housing environment when mother was an elementary school child-aged; Model 3: Model 1 (six allergic symptoms integrated into any allergic symptoms) + housing environment when mother was an elementary school child-aged and psychosomatic state (nervousness). Text in parentheses reflects case groups. ^a^ORs and 95% CIs for six-level linear variables were calculated using one unit of change. ^b^The six levels were (1) not at all (0%), (2) slight (20%), (3) somewhat (40%), (4) moderate (60%), (5) often (80%), and (6) always (100%)

Cold sensitivity was the only variable of physical constitution that significantly related to CI in all tested models. Unregulated ingestion of meals was significantly associated with CI in all tested models. Bronchial asthma and any allergies in the past medical history were significantly associated with CI in all tested models. Living with an indoor cat during childhood was the only variable in the housing environment that significantly associated with CI in model 2. The association with the presence of indoor cat was no longer significant after testing with personal factors, past medical history, and psychosomatic state during past month (model 3). The univariate analysis showed that the current presence of an indoor cat was not significantly associated with CI.

### Mother– and/or father–child relationships

Table 5 provides ORs for the medical history of the 3-year-old children according to the current CI of the mothers. The associations with rheumatoid arthritis, gastric ulcer, MCS, and sick building syndrome were not analyzed because of the small number of cases. Crude OR was adjusted for the gender and birth order of the child, age and employment status of the mother, and drinking habits and smoking history of the mother. The current CI of the mother was significantly associated with allergic rhinitis and bronchial asthma of the child, even after adjusting for those variables, and was significantly associated with chronic bronchitis of the child, after adjusting for the variables.

**Table 5:** Odds ratios for medical histories of 3-year-old children according to chemical intolerance of mothers

Medical history^a^	Crude OR (95% CI)^b^	Adjusted OR (95% CI)^c^
Allergies		
Hay fever	2.27 (0.52–9.89)	1.12 (0.15–8.56)
Allergic rhinitis	2.47 (1.31–4.68)**	2.32 (1.19–4.53)*
Allergic conjunctivitis	1.40 (0.43–4.59)	1.50 (0.45–4.96)
Alimentary allergy	1.16 (0.62–2.15)	1.02 (0.52–2.02)
Medication allergy	2.88 (0.65–12.78)	1.53 (0.19–12.11)
Atopic dermatitis	1.42 (0.76–2.66)	1.36 (0.70–2.64)
Any allergies^d^	1.60 (1.02–2.52)*	1.42 (0.87–2.31)
Bronchial asthma	3.17 (1.79–5.61)***	3.66 (2.00–6.69)***
Empyema, sinusitis	1.75 (0.79–3.90)	1.58 (0.67–3.77)
Chronic bronchitis	3.44 (0.99–11.84)	3.69 (1.04–13.03)*

^a^Not applicable in rheumatoid arthritis, gastric ulcer, multiple chemical sensitivity, and sick building syndrome due to the small numbers. ^b^Values are expressed as crude odds ratios (95% CI) for 2044 participants with complete data. ^c^Values are expressed as adjusted odds ratios (95% CI) for 2044 participants with complete data (adjusted for gender, birth order, age and employment status of mother, drinking habits of mother, and smoking history of mother). ^d^At least one of six allergies (hay fever, allergic rhinitis, allergic conjunctivitis, alimentary allergy, medication allergy, atopic dermatitis) reported by participants. Significant at *p < 0.05, **p < 0.01, ***p < 0.001. *CI* confidential interval, *OR* odds ratio

We additionally tested the current CI of the 3-year-old children and current CI and past medical history of the mother and father, although only a few variables were tested because of the small number of children with CI. The univariate associations between current CI of the 3-year-old children and all personal and other variables are listed in Additional file 1: Table S1, and the subsequent results of the multiple logistic regression analyses are shown in Additional file 1: Table S2. The CI of the mother was significantly associated with the CI of the child in all tested models. Chronic bronchitis of the mother was significantly associated with the CI of the child (model 1), but after adjusting for the medical history of father, the association with chronic bronchitis was no longer significant. Gastric ulcer of the father was significantly associated with the CI of the child in all tested models.

## Discussion

In the present study, the prevalence of CI in mothers and 3-year-old children is 4.5% and 0.25%, respectively. The prevalence of self-reported CI is rather high, 9 to 33%, whereas it drops to 0.5 to 6.3% when fulfilling the clinical criteria for CI [24]. A study by Black et al. using the same questionnaire and definition used in our study reported that the prevalence of CI was 2.6% in general military personnel (10.4% females, 89.6% males) [28]. Given that a preponderance of women among CI sufferers is almost consistently reported in population-based studies [16] and that a Japanese population-based study reported that the CI prevalence in females was approximately twofold higher than in males [1], the prevalence in mothers in this study suggests a similar level to that of the study by Black et al. [28]. Although one study reported that the age distribution of CI prevalence in teenagers was almost one third of that in young people in their 20s [20], to our best knowledge, the prevalence of CI in children under 10 years of age has not been reported. The present study shows that the CI prevalence in 3-year-old children is almost one eighteenth that of their mothers.

In the present study, cold sensitivity in mothers is significantly associated with CI in mothers. Cold sensitivity is a common symptom in Japanese women. Interestingly, cold sensitivity is observed in approximately one half of Japanese women, and it can lead to a decreased quality of life [36]. To the best of our knowledge, our result is the first reporting on this association among the studies looking at CI including MCS and IEI. Cold sensitivity is an elusive condition that has previously been defined as an exaggerated or abnormal reaction to cold exposure causing discomfort, including symptoms involving the central nervous system, respiratory, gastrointestinal, musculoskeletal, and peripheral vascular problems, that can be accompanied by pain, numbness, stiffness, weakness, and swelling. The pathophysiological mechanisms are not fully elucidated but seem to involve a multifactorial etiology, including neural, vascular, and humoral aspects; disturbance of the autonomic system; lifestyle; and psychological stress [36, 37]. The etiology of peripheral coldness is also not well established, but it may be associated with decreased peripheral blood flow [38]. Symptoms of fibromyalgia can arise from peripheral influences of the autonomic nervous system in response to aversive mood states (e.g., anxiety, fear, sorrow, and depression) [39] that are relatively common chronic conditions with CI [40, 41]. Stress, when chronic, results in cardiovascular dysfunction with reduced peripheral blood flow due to vasoconstriction [42, 43]. The resting levels of peripheral vasoconstriction are particularly high for women relative to men [44–46], and CI is primarily a disorder in women [1, 16]. Therefore, understanding the association of CI with cold sensitivity, especially peripheral influences of the autonomic nervous system, may provide further insights into the underlying mechanisms of CI.

The present study indicates that asthma and allergies are significantly associated with CI, which is consistent with several previous studies [1, 24, 47, 48]. Unregulated ingestion of meals is significantly associated with CI. A recent study reported that a high percentage of patients with CI had a poor nutritional status, low muscle strength, and decreased muscle mass [49]. Whether these changes are the cause or a consequence of CI, however, remains unclear.

In the present study, the presence of an indoor cat during childhood is significantly associated with current CI in mothers, but the association is weak compared with cold sensitivity and past asthma and allergies. In allergic diseases, systematic reviews suggest that the overall cumulative evidence shows no increase in risk of allergic disease from early exposure to pet allergens [26], with a possible decreased risk of asthma associated with cat allergen exposure in one study [50]. Therefore, early exposure to pet allergens is not likely to be associated with the development of CI. Rather, early exposure to odor or irritant substances emitted from excrement of pets, including body odor, urine, or feces, may be associated with the development of CI.

The present study indicates that current CI of mothers is significantly associated with allergic rhinitis, bronchial asthma, and chronic bronchitis of 3-year-old children. A study reported that a high percentage of patients with CI had a poor nutritional status [49]. In the present study, unregulated ingestion of meals is significantly associated with CI. Nutrition in early life is associated with risk of asthma and allergic disease [51–53]. The nutritional status in mothers with CI may be affecting the respiratory hypersensitivity of the children. Otherwise, poor housing conditions due to lower socioeconomic status may increase the exposure to indoor environmental allergens associated with asthma and rhinitis, including microbial agents, house dust mites, pests, or molds, during early life [26]. The CI of mothers is significantly associated with the CI of children in all tested models, although only a few variables were tested because of the small number of 3-year-old children with CI. One study suggested that exposure to tobacco smoke, disinfectants, or volatile organic compounds in the first year of life contributed to an enhanced risk for the development of wheezing symptoms within the second year of life [54]. However, patients with CI attempted to avoid exposure to odorous chemicals as much as possible in their daily life [13–15]. Therefore, further investigations on these associations in the maternal lifestyle and housing environment during the prenatal period in mothers and in the early life of the children are needed.

A recent study reported that avoidance behavior related to odorous and/or pungent chemicals has increased in persons with CI. The environmental risk factors related to CI have become diversified in the past decade due to lifestyle changes and the development of novel household products and building materials [55]. Therefore, the endogenous and/or exogenous causes of CI and the underlying mechanisms of CI need to be identified. This will be very important in preventing CI and improving the quality of life for those experiencing CI. Our findings can provide novel and progressive insights on these issues in CI.

There are some limitations associated with our study. First, because the study design is a cross-sectional retrospective, it is difficult to determine the cause and effect of temporal relationships. Second, this study is based on self-reported responses to a questionnaire. The environmental reports from the participants are highly subjective, and the resulting inaccuracies may result in bias. This is also true of the subjective, self-reported CI assessments used in this study, despite the use of a well-known questionnaire that has been developed by a panel of physician experts. Third, although we attempted to discriminate between current CI of 3-year-old children and current CI and past medical history of the mother and father, only a few variables could be tested because of the small number of 3-year-old children with CI. Further large-scale study is required to examine the factors related to development of CI in 3-year-old children.

## Conclusions

We estimate that the prevalence of CI in 3-year-old children is almost one eighteenth of that experienced by their mothers. Our finding suggests that inherent physical constitution, especially cold sensitivity, is associated with the risk of acquiring CI. We also suggest that the presence of an indoor cat during childhood may be associated with a risk of acquiring CI. Children of mothers with CI have a possible risk of respiratory hypersensitivity or inflammation. Understanding the association of CI with cold sensitivity, resulting in peripheral influences of the autonomic nervous system, may provide further insights into the underlying mechanism of CI. The relationships between the CI of mothers and respiratory hypersensitivity or inflammation and the CI of their children are interesting. Characteristics of inherent physical constitution may affect those relationships. Further investigation of the inherent physical constitution and background environmental factors associated with risk of acquiring CI, especially during the prenatal period in the mother and in the early life of the children, is required. The impact of having mothers with chemical intolerance on the health of children also requires further study.

## Additional file

Additional file 1: **Table S1.** Univariate analysis for the chemical intolerance of 3-year-old children. **Table S2.** Multivariable analysis for the chemical intolerance of 3-year-old children. (DOCX 31 kb)
