# Supplementary material for: Background factors of chemical intolerance and parent–child relationships
Source: Environ Health Prev Med. 2018 Oct 24;23:52. doi: 10.1186/s12199-018-0743-y (PMC6201541; doi:10.1186/s12199-018-0743-y)
Supplement: Supplementary file 1 — Table S1. Univariate analysis for the chemical intolerance of 3-year-old children. Table S2. Multivariable analysis for the chemical intolerance of 3-year-old children. (DOCX 31 kb) [file 12199_2018_743_MOESM1_ESM.docx]

Online supporting information for the following article

Background factors of chemical intolerance and parent–child relationships

**Table S1. Univariate analysis for the chemical intolerance of three-year-old children.**

| Variables | Crude OR (95% CI) |
| --- | --- |
| Personal factors of three-year-old children |  |
| Gender (female) | 0.28 (0.03–2.47) |
| Birth order (second or after) | 0.25 (0.03–2.19) |
| Eating regular meals (with) | – |
|  |  |
| Personal factors of mothers |  |
| Age  18–29  30–39  40–49  *p* for trend | Ref.  –  –  0.657 |
| Body mass index  Low weight  Normal weight  Obesity  *p* for trend | Ref.  0.69 (0.07–6.66)  1.91 (0.12–30.71)  0.676 |
| Employment  Homemaker  Part-time employed  Employed  Others | Ref.  –  –  – |
| Smoking history (with) | – |
| Drinking habits  Never  1–3 days a week  4–6 days a week  Everyday  *p* for trend | Ref.  1.81 (0.30–10.89)  –  –  0.935 |
| Regular exercise (with) | 2.00 (0.33–12.00) |
| Eating regular meals (with) | 0.66 (0.07–5.95) |
| Sweaty (with) | 1.13 (0.19–6.76) |
| Cold sensitivity (with) | – |
|  |  |
| Past medical history |  |
| Mothers |  |
| Chemical intolerance | 93.68 (10.35–847.62) ^***^ |
| Allergies |  |
| Hay fever | – |
| Allergic rhinitis | – |
| Allergic conjunctivitis | – |
| Alimentary allergy | – |
| Medication allergy | – |
| Atopic dermatitis | – |
| Any allergies*^b^* | – |
| Bronchial asthma | 4.71 (0.52–42.53) |
| Empyema, sinusitis | – |
| Chronic bronchitis | 35.18 (3.70–334.93)^**^ |
| Rheumatoid arthritis | – |
| Gastric ulcer | – |
| Multiple chemical sensitivity | – |
| Sick building syndrome | – |
|  |  |
| Fathers |  |
| Allergies |  |
| Hay fever | 1.75 (0.20–15.72) |
| Allergic rhinitis | 2.51 (0.28–22.54) |
| Allergic conjunctivitis | – |
| Alimentary allergy | 23.37 (2.51–218.00)^**^ |
| Medication allergy | – |
| Atopic dermatitis | – |
| Any allergies*^b^* | 0.87 (0.10–7.78) |
| Bronchial asthma | – |
| Empyema, sinusitis | 3.92 (0.44–35.33) |
| Chronic bronchitis | – |
| Rheumatoid arthritis | – |
| Gastric ulcer | 22.14 (3.63–135.02)^***^ |
| Multiple chemical sensitivity | – |
| Sick building syndrome | – |
|  |  |
| Three-year-old children |  |
| Allergies |  |
| Hay fever | 24.54 (2.63–229.37)^**^ |
| Allergic rhinitis | 3.88 (0.43–35.00) |
| Allergic conjunctivitis | – |
| Alimentary allergy | 5.16 (0.86–31.03) |
| Medication allergy | – |
| Atopic dermatitis | 2.31 (0.26–20.73) |
| Any allergies*^b^* | 2.25 (0.37–13.48) |
| Bronchial asthma | 9.43 (1.56–56.90)^*^ |
| Empyema, sinusitis | 5.14 (0.57–46.44) |
| Chronic bronchitis | – |
| Rheumatoid arthritis | – |
| Gastric ulcer | – |
| Multiple chemical sensitivity | – |
| Sick building syndrome | – |
|  |  |
| Housing environment in elementary school-aged child in mothers |  |
| Type of housing  Reinforced  Wooden  Combined with reinforced and wooden | Ref.  1.32 (0.15–11.86)  – |
| Floor material |  |
| Wood | 2.87 (0.48–17.24) |
| Japanese tatami | – |
| Carpet | 0.96 (0.16–5.78) |
| Plastics | – |
| Spray of pesticide indoors (with) | 0.82 (0.14–4.92) |
| Use of mosquito coil indoors (with) | – |
| Indoor pet parenting in the first decade of life |  |
| Dog | 3.52 (0.59–21.15) |
| Cat | – |
|  |  |
| Housing environment (current) |  |
| Type of housing  Reinforced  Wooden  Combined with reinforced and wooden | Ref.  0.54 (0.09–3.25)  – |
| Floor material |  |
| Wood | 2.22 (0.25–19.90) |
| Japanese tatami | 1.48 (0.25–8.85) |
| Carpet | 1.35 (0.23–8.12) |
| Plastics | – |
| Spray of pesticide indoors (with) | 0.82 (0.14–4.91) |
| Use of mosquito coil indoors (with) | – |
| Indoor pet parenting |  |
| Dog | – |
| Cat | – |
| Passive smoking (with) | 0.67 (0.11–4.00) |
|  |  |
| Psychosomatic state past month in mothers*^a,b^* |  |
| Nervous | 0.84 (0.37–1.94) |
| Peaceful | 0.97 (0.52–1.84) |
| Depressed feeling | 0.65 (0.23–1.85) |
| Happiness feeling | 1.62 (0.64–4.09) |
| Discouraged | 1.19 (0.57–2.48) |

Values are expressed as crude odds ratios (95% CI) for 2044 participants with complete data. Ref. = referent. Significant at ^*^ *p* < 0.05, ^**^ *p* < 0.01, ^***^ *p* < 0.001. Text in parentheses reflects case groups. Horizontal lines were expressed as the variable not analyzed due to the small numbers. *^a^* ORs and 95% CIs for six-level linear variables were calculated using one unit of change. *^b^* The six levels were 1) not at all (0%), 2) slight (20%), 3) somewhat (40%), 4) moderate (60%), 5) often (80%), and 6) always (100%).

**Table S2. Multivariable analysis for the chemical intolerance of three-year-old children.**

| Variables | Model 1 | Model 2 | Model 3 | Model 4 | Model 5 |
| --- | --- | --- | --- | --- | --- |
| Past medical history |  |  |  |  |  |
| Mothers |  |  |  |  |  |
| Chemical intolerance | 173.5 (12.9–2327.1) ^***^ |  | 261.9 (13.9–4947.1) ^***^ |  | 885.2 (9.13–85851.5) ^***^ |
| Chronic bronchitis | 97.4 (4.20–2259.0)^**^ |  | 14.4 (0.31–681.0) |  | 1.66 (0.02–130.6) |
|  |  |  |  |  |  |
| Fathers |  |  |  |  |  |
| Alimentary allergy |  | 18.6 (1.47–234.6)^*^ | *^a^* |  | *^a^* |
| Gastric ulcer |  | 17.2 (2.56–115.3)^**^ | 42.7 (2.25–808.3)^*^ |  | 100.6 (1.78–5691.5)^*^ |
|  |  |  |  |  |  |
| Three-year-old children |  |  |  |  |  |
| Hay fever |  |  |  | 11.45 (1.01–129.92)^*^ | 45.35 (0.44–4652.2) |
| Alimentary allergy |  |  |  | 1.97 (0.26–14.61) | 1.21 (0.05–28.65) |
| Bronchial asthma |  |  |  | 6.84 (1.04–45.10)^*^ | 2.47 (0.17–35.56) |
| Empyema, sinusitis |  |  |  | 3.35 (0.34–33.21) | 4.77 (0.23–100.0) |

Values are expressed as adjusted odds ratios (95% confidential interval) for 2044 participants with complete data. Ref. = referent. Significant at ^*^ *p* < 0.05, ^**^ *p* < 0.01, ^***^ *p* < 0.001. Text in parentheses reflects case groups. *^a^* Alimentary allergy of father was not included in the model because of incomplete model fitting. The predictor variables of chemical intolerance of three-year-old children were selected from possible risk factors, possible confounders, and multicollinearity test (*r* < 0.5). Model 1: gender, birth order, and past medical history of mother; Model 2: gender, birth order, and past medical history of father; Model 3: Model 1 + past medical history of father; Model 4: gender, birth order, and past medical history of three-year-old children; Model 5: Model 3 + Model 4.
